# Distinct risks of exacerbation and lung function decline between never-smokers and ever-smokers with COPD

**DOI:** 10.1186/s12890-025-03604-1

**Published:** 2025-03-28

**Authors:** Heemoon Park, Soo Min Jo, Kwang Nam Jin, Hyo Jin Lee, Hyun Woo Lee, Tae Yun Park, Eun Young Heo, Deog Kyeom Kim, Jung-Kyu Lee

**Affiliations:** 1 Division of Pulmonary and Critical Care Medicine, Department of Internal Medicine, Seoul Metropolitan Government-Seoul National University Boramae Medical Center, 20 Boramaero-5-Gil, Dongjak-Gu, Seoul, 07061 Republic of Korea; 2 Division of Pulmonary and Critical care medicine, Department of Internal medicine, Dongguk University Ilsan Hospital, Goyang, Republic of Korea; 3 Department of Radiology, Seoul Metropolitan Government-Seoul National University Boramae Medical Center, Seoul, Republic of Korea

**Keywords:** COPD, Never-smoker, Exacerbation, Lung function

## Abstract

**Background:**

Chronic obstructive pulmonary disease (COPD) can occur in patients without a history of smoking, which is a strong risk factor for COPD. However, few studies have focused on the prognosis of never-smokers with COPD. We investigated the difference of the longitudinal clinical outcomes between never-smokers and ever-smokers with COPD.

**Methods:**

We retrospectively analyzed patients with COPD who underwent chest computed tomography and longitudinal lung function tests from January 2013 to December 2020. We classified patients according to smoking status and examined their histories of acute exacerbation and long-term changes in lung function.

**Results:**

Among 583 patients with COPD, 75 (12.9%) had no smoking history. These never-smokers with COPD were predominantly women; they had a lower forced vital capacity and a higher prevalence of asthma, history of tuberculosis, tuberculosis-destroyed lung, and bronchiectasis, but a lower prevalence of emphysema, relative to ever-smokers with COPD. Never-smokers with COPD had significantly lower risks of subsequent moderate to severe exacerbation (β ± standard error, − 0.4 ± 0.12; *P* = 0.001), any exacerbation (adjusted odds ratio, 0.46; 95% confidence interval, 0.26 − 0.8; *P* = 0.006), and frequent exacerbation (adjusted odds ratio, 0.28; 95% confidence interval, 0.09 − 0.89; *P* = 0.03) than ever-smokers with COPD. Never-smokers with COPD also showed significantly slower annual decline of forced expiratory volume in 1 s than ever-smokers with COPD (− 15.7 ± 4.7 vs. −30.4 ± 2.9 mL, respectively; *P* = 0.03).

**Conclusions:**

Never-smokers with COPD had significantly fewer acute exacerbations and slower decline of lung function than ever-smokers with COPD during longitudinal follow-up.

**Supplementary information:**

The online version contains supplementary material available at 10.1186/s12890-025-03604-1.

## Introduction

The development of chronic obstructive pulmonary disease (COPD) is multifactorial, influenced by both genetic and environmental risk factors [1]. Smoking, the strongest risk factor for COPD, plays a key role in its onset and progression [2]. However, smoking is not the only cause of COPD, and the proportion of never-smokers among patients with COPD has been reported to be approximately 25–45%, based on a systematic review of existing literature [3]. Various environmental, disease-related, or host-related factors may contribute to the development of COPD in never-smokers, including indoor and outdoor air pollution, occupational exposure, tuberculosis (TB) and other respiratory tract infections, asthma, and socioeconomic factors (e.g., low educational attainment or poor nutrition) [4–8].

Recent studies have suggested that COPD patients without a smoking history have different phenotypes and clinical courses compared to COPD patients with a smoking history. In a study using the Copenhagen General Population Study cohort to compare never-smoker COPD, ever-smoker COPD, and non-COPD patients, it was found that never-smoker COPD patients had fewer respiratory symptoms and lower levels of inflammatory markers compared to ever-smoker COPD patients [9]. According to a study by Salvi et al., never-smoker COPD patients had a higher proportion of females, less emphysema, and characteristic small airway obstruction compared to ever-smoker COPD patients, as well as significantly less decline in lung function over a one-year period [10]. These findings suggest that never-smoker COPD has a lower long-term mortality risk compared to ever-smoker COPD but a significantly higher risk compared to never-smokers without COPD, indicating that lung-related morbidity and mortality can still be substantial in never-smoker COPD patients.

In the present study, we examined the proportion of never-smokers among patients with COPD and evaluated the distinct characteristics and long-term clinical outcomes of never-smokers with COPD compared to ever-smokers with COPD.

## Methods

### Study population

This retrospective study was conducted at Seoul Metropolitan Government–Seoul National University Boramae Medical Center, a tertiary referral hospital. We included patients who had been diagnosed with COPD according to spirometric criteria (post-bronchodilator forced expiratory volume in 1 s (FEV_1_)/forced vital capacity (FVC) < 0.7) and undergone at least one chest computed tomography (CT) scan and at least three longitudinal lung function tests from January 2013 to December 2020. Patients with a follow-up period of < 12 months were excluded from the study.

The study population was classified into three groups according to smoking status: never-smokers, ex-smokers, and current smokers. Patients with no prior smoking history at the time of enrollment were classified as never-smokers, while those who were still smoking were classified as current smokers. Patients who had maintained smoking cessation for > 6 months prior to study enrollment were classified as ex-smokers, and the combination of ex-smokers and current smokers was defined as ever-smokers. Comorbidity severities were assessed using the Charlson comorbidity index [11]. We investigated each patient’s inhaler usage during the study period, including inhaled long-acting muscarinic antagonists, inhaled long-acting β2-agonists, and inhaled corticosteroids. Patients with a history of continuous inhaler use for at least 3 months were regarded as inhaler users. The inhaler medication possession ratio (MPR) was defined as the ratio of the duration for which inhalers were used by each patient during the follow-up period. Subgroup analysis according to airflow limitation severity was performed by classifying the study population into four Global Initiative for Chronic Obstructive Lung Disease (GOLD) spirometric grades based on the recent GOLD document: grade 1, FEV_1_ ≥ 80% predicted; grade 2, 50% ≤ FEV_1_ < 80% predicted; grade 3, 30% ≤ FEV_1_ < 50% predicted; and grade 4, FEV_1_ < 30% predicted [12]. Bronchodilator response (BDR) was defined as a ≥ 12% and ≥ 200-mL increase in either FEV_1_ and/or FVC from baseline after short-acting β2-agonist therapy, as confirmed in the initial spirometric evaluation [13].

The Institutional Review Board of Seoul Metropolitan Government–Seoul National University Boramae Medical Center approved this study (approval no. 30-2021-26) and waived the requirement for informed consent because of the retrospective nature of the study and the lack of participant intervention or interaction. The study was conducted in accordance with the principles stated in the Declaration of Helsinki.

### Outcomes

The primary outcome was the annual rate of moderate to severe acute exacerbations of COPD. This study was a retrospective study conducted over multiple years, and unlike prospective studies, it was not possible to control the baseline status of each participant at the start of their study period. Therefore, we selected annual incidence as an indicator to assess the average occurrence of acute exacerbations over the study period. Additionally, to distinguish stable patients who experienced no acute exacerbations over several years from those who did, we used ‘any exacerbation’ as a variable. Conversely, to identify and analyze high-risk patients who experienced repeated exacerbations, we selected the variable ‘frequent exacerbator’. Acute exacerbation was defined as an acute event characterized by worsening of respiratory symptoms requiring additional treatment, and acute exacerbation severity was classified as moderate (event treated with oral antibiotics and/or systemic steroids) or severe (event requiring hospitalization or emergency room visit). We separately investigated the occurrences of moderate exacerbations and severe acute exacerbations among the study participants during the study period. Moderate-to-severe exacerbations were calculated to include both moderate and severe exacerbations. Frequent exacerbation was defined as two or more moderate exacerbations, or one or more severe exacerbations, within 1 year.

The secondary outcome was the annual change in lung function as determined by longitudinal changes in FEV_1_, FVC, FEV_1_/FVC ratio, and diffusing capacity of the lung for carbon monoxide (D_LCO_).

### Radiological findings

We analyzed baseline chest CT findings during the study period to determine their associations with COPD onset and progression. Chest CT findings were visually assessed by three readers: two pulmonologists (S.M.J. and J.K.L.) reviewed the images under the supervision and consensus reading of one chest radiologist (K.N.J.). We confirmed the presence of emphysema, TB-destroyed lung, bronchiectasis, and interstitial lung disease (ILD), using chest CT images. TB-destroyed lung was defined as parenchymal destruction involving one or more lobes as a result of past TB infection [14]. Bronchiectasis was defined as bronchial dilatation compared with adjacent pulmonary arteries, lack of bronchial tapering, and presence of bronchi within 1 cm of the pleural surface [15]. ILD was defined as chronic interstitial disease characterized by airspace opacification, reticulation, and honeycombing [16].

### Statistical analysis

Data are presented as mean ± standard deviation for continuous variables and as number (percentage) for categorical variables. The chi-square test, Fisher’s exact test, and t-test were performed to compare baseline characteristics and history of acute exacerbation between the groups, and logistic regression was performed to explore factors associated with COPD. Binary logistic regression analysis was performed to explore factors associated with never-smokes with patients with COPD. The indicators related to lung function have strong multicollinearity, so during the statistical analysis process, we selected the most representative indicators in terms of significance to include in the prediction model, forming the model based on those with the most significant impact. In this process, inhaler use and the medication possession ratio were excluded from the analysis model for factors associated with never-smoker status, considering their lower priority in terms of statistical significance. Linear regression and logistic regression were used to analyze the risks of acute exacerbation and frequent exacerbation. We developed a model to predict the occurrence of acute exacerbations in the study population and identified variables that showed significant associations during this process. In analyzing the association between smoking status and acute exacerbations, these variables were used to enhance the validity of the results. Consequently, the multivariable analysis for the risk of acute exacerbation was adjusted for FEV_1_, D_LCO_, blood neutrophil-to-lymphocyte ratio, ILD, and inhaler MPR. Linear mixed regression analysis was performed to analyze longitudinal changes in lung function according to smoking status. The multivariable model of lung function was adjusted for age, sex, height, and baseline lung function. Odds ratios (ORs) and adjusted ORs (aORs) were generated with 95% confidence intervals (CIs). *P*-values < 0.05 were considered statistically significant. Statistical analyses were conducted using SPSS software version 29.0 (IBM Corp., Armonk, NY, USA).

## Results

### Baseline characteristics

**Fig. 1:**
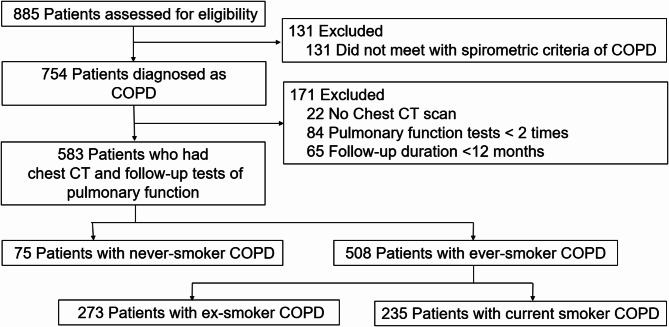
Flow diagram of study population. COPD, chronic obstructive pulmonary disease; CT, computed tomography

Among 885 patients assessed for eligibility, 583 were finally included in the primary analysis after application of the inclusion and exclusion criteria (Fig. 1). Among these 583 patients, 75 (12.9%) were never-smokers, 273 (46.8%) were ex-smokers, and 235 (40.3%) were current smokers. The mean patient age and follow-up duration were 66.2 and 5.3 years, respectively.

The baseline characteristics of the study population are presented in Table 1. Compared with ever-smokers who had COPD, never-smokers with COPD were characterized by more female sex (50.7% vs. 3.3%), higher prevalence of history of TB (44% vs. 24%) and physician-diagnosed asthma (37.3% vs. 24.4%). With regard to lung function, never-smokers had a significantly lower FVC and forced expiratory flow between 25% and 75% of vital capacity (FEF_25%–75%_), lower proportion of BDR, and higher D_LCO_. There were no differences in the blood neutrophil-to-lymphocyte ratio or presence of eosinophilia. Regarding the baseline CT findings, never-smokers had significantly less emphysema but more TB-destroyed lung and bronchiectasis relative to ever-smokers. The proportion of inhaler users was higher among never-smokers, but there was no difference in the inhaler MPR between never-smokers and ever-smokers.

**Table 1:** Baseline and clinical characteristics of the study population

	Never-smoker COPD (*n* = 75)	Ever-smoker COPD (*n* = 508)	*P*-value
Follow-up duration, year	5.9 ± 3.8	5.2 ± 3.2	0.149*
Age, years	64.8 ± 11.3	66.4 ± 9.1	0.259*
Female sex, *n* (%)	38 (50.7)	17 (3.3)	< 0.001†
Body mass index, kg/m^2^	22.3 ± 3.2	22.3 ± 3.4	0.778*
Smoking intensity, pack-years	0 ± 0	42.4 ± 23.7	< 0.001*
Charlson comorbidity index	1.53 ± 1.02	1.56 ± 1.05	0.701*
History of tuberculosis, *n* (%)	33 (44)	122 (24)	< 0.001†
History of NTM lung disease, *n* (%)	3 (4)	6 (1.2)	0.065†
Physician-diagnosed asthma, *n* (%)	28 (37.3)	124 (24.4)	0.017†
Baseline lung function			
FEV_1_, L	1.4 ± 0.5	1.7 ± 0.6	< 0.001*
FEV_1_, % predicted	62.3 ± 18	66.5 ± 19	0.059*
FVC, L	2.5 ± 0.7	3.3 ± 0.8	< 0.001*
FVC % predicted	78.8 ± 17.2	89.6 ± 17.7	< 0.001*
FEV_1_/FVC, %	55.3 ± 10	51.4 ± 11.6	0.007*
FEF_25%–75%_, % predicted	24.9 ± 9.8	27.9 ± 11.6	0.039*
D_LCO_, mL/mmHg/min	14.2 ± 4.1	13.5 ± 4.3	0.131*
D_LCO_, % predicted	85.6 ± 22.7	81.1 ± 21.2	0.033*
Positive bronchodilator response, *n* (%)	1 (1.3)	68 (13.4)	0.003†
Laboratory findings			
Blood neutrophil-to-lymphocyte ratio	4.6 ± 9.2	3.6 ± 3.6	0.286*
Blood eosinophil count, × 10^3^/L	221.6 ± 274.8	224.8 ± 234	0.408*
Blood eosinophil count of > 300 × 10^3^/L, *n* (%)	14 (18.7)	113 (22.2)	0.484†
Baseline CT findings			
Emphysema, *n* (%)	15 (20)	406 (79.9)	< 0.001†
Tuberculosis-destroyed lung, *n* (%)	18 (24)	41 (8.1)	< 0.001†
Bronchiectasis, *n* (%)	52 (69.3)	226 (44.5)	< 0.001†
Interstitial lung disease, *n* (%)	1 (1.3)	13 (2.6)	0.518†
Inhaler use, *n* (%)	74 (98.7)	467 (91.9)	0.035†
Inhaler medication possession ratio	0.7 ± 0.3	0.6 ± 0.4	0.421*

Data are presented as mean ± standard deviation or *n* (%)

*P-values were calculated using the **t-test** for continuous variables

†*P*-values were calculated using the **chi-square test** (or Fisher’s exact test where applicable) for categorical variables

COPD = chronic obstructive pulmonary disease; CT = computed tomography; D_LCO_ = diffusing capacity of the lungs for carbon monoxide; FEF_25%–75%_ = forced expiratory flow between 25% and 75% of vital capacity; FEV_1_ = forced expiratory volume in 1 s; FVC = forced vital capacity; NTM = nontuberculous mycobacteria

These distinctive between-group differences remained consistent when analyses were conducted by stratifying the ever-smoker group into ex-smokers and current smokers (Table S1).

### Factors associated with never-smokers compared to ever-smokers among patients with COPD

We analyzed factors significantly associated with never-smokers compared to ever-smokers among patients with COPD, considering differences in baseline characteristics (Table 2). Univariable analysis showed that never-smoker COPD was positively associated with female sex, absence of emphysema, TB-destroyed lung, bronchiectasis, history of tuberculosis, and physician-diagnosed asthma. In contrast, never-smokers COPD was inversely associated with FVC and positive BDR. According to multivariable analysis, never-smokers in patients with COPD were significantly associated with female sex (aOR, 15.4; 95% CI, 6.81 − 34.9; *P* < 0.001), absence of emphysema (aOR, 9.01; 95% CI, 0.05 − 0.21; *P* < 0.001), and TB-destroyed lung (aOR, 4.14; 95% CI, 1.56 − 11.2; *P* = 0.005); they were inversely associated with positive BDR (aOR, 0.12; 95% CI, 0.02 − 1.01; *P* = 0.047).

**Table 2:** Factors associated with never-smokers compared to ever-smokers among patients with COPD

Variable	Univariable		Multivariable	
	OR (95% CI)	*P*-value	aOR (95% CI)*	*P*-value
Female sex	29.7 (15.3–57.5)	< 0.001	15.4 (6.81–34.9)	< 0.001
History of tuberculosis	2.49 (1.51–4.1)	< 0.001	1.25 (0.59–2.57)	0.551
Physician-diagnosed asthma	1.85 (1.11–3.07)	0.019	1.50 (0.71–2.97)	0.269
FVC % predicted	0.97 (0.96–0.99)	< 0.001	0.99 (0.41–1.05)	0.199
Positive bronchodilator response	0.09 (0.01–0.64)	0.016	0.12 (0.02–0.98)	0.047
Absence of emphysema	15.9 (8.69–29.2)	< 0.001	9.01 (0.05–0.21)	< 0.001
Tuberculosis-destroyed lung	3.60 (1.94–6.68)	< 0.001	4.14 (1.56–11.2)	0.005
Bronchiectasis	2.82 (1.68–4.75)	< 0.001	1.86 (0.89–3.87)	0.099

*Adjusted for all variables used in the univariable analysis

OR = odds ratio; aOR = adjusted odds ratio; CI = confidence interval; FVC = forced vital capacity

### Incidence and risk of subsequent acute exacerbation according to smoking status

We investigated the incidence of subsequent acute exacerbation during the follow-up period. The mean annual incidences of moderate to severe acute exacerbation in never-smokers and ever-smokers were 0.43 ± 0.64 and 0.65 ± 1.07 times, respectively (Table 3). Although the differences were not statistically significant, never-smokers had lower annual incidences of moderate and severe acute exacerbations and lower incidences of exacerbations and frequent exacerbators compared with ever-smokers.

**Table 3:** History of acute exacerbations according to smoking status

	Never-smoker (*n* = 75)	Ever-smoker (*n* = 508)	*P*-value*	Ex-smoker (*n* = 273)	Current smoker (*n* = 235)	*P*-value†
Annual incidence of acute exacerbations by severity, time/year						
Moderate	0.22 ± 0.4	0.38 ± 0.67	0.078	0.44 ± 0.74	0.32 ± 0.58	0.078
Severe	0.21 ± 0.39	0.26 ± 0.67	0.592	0.29 ± 0.71	0.23 ± 0.62	0.574
Moderate to severe	0.43 ± 0.64	0.65 ± 1.07	0.198	0.72 ± 1.17	0.55 ± 0.93	0.132
Any exacerbation, *n* (%)	41 (54.7)	301 (59.3)	0.452	168 (61.5)	133 (56.6)	0.399
Frequent exacerbator, *n* (%)	4 (5.3)	47 (9.3)	0.288	32 (11.7)	15 (6.4)	0.056

Data are presented as mean ± standard deviation or *n* (%)

*Comparison of never-smokers and ever-smokers with COPD

†Comparison of never-smokers, ex-smokers, and current smokers with COPD

In the multivariable analysis adjusted for FEV_1_, D_LCO_, blood neutrophil-to-lymphocyte ratio, ILD, and inhaler MPR, never-smokers had significantly lower risks of moderate, severe, and moderate to severe acute exacerbations compared with ever-smokers (Table 4). Never-smokers had a 64% lower risk of subsequent acute exacerbations and a 72% lower risk of frequent exacerbations during the follow-up period compared with ever-smokers (Table 5).

**Table 4:** Risk of subsequent acute exacerbation according to smoking status, estimated through annualized occurrences of acute exacerbations

	Moderate exacerbation		Severe exacerbation		Moderate-to-severe exacerbation	
	β ± SE	*P*-value*	β ± SE	*P*-value*	β ± SE	*P*-value*
Never-smoker (vs. ever-smoker)	−0.23 ± 0.08	0.004	−0.17 ± 0.08	0.029	−0.4 ± 0.12	0.001
FEV_1_, L	−0.19 ± 0.06	0.001	−0.29 ± 0.06	< 0.001	−0.48 ± 0.09	< 0.001
D_LCO_, mL/mmHg/min	0 ± 0.01	0.995	−0.02 ± 0.01	0.006	−0.02 ± 0.01	0.088
Blood neutrophil-to-lymphocyte ratio	0 ± 0.01	0.846	0.03 ± 0.01	< 0.001	0.03 ± 0.01	0.001
Interstitial lung disease	0.37 ± 0.17	0.029	0.13 ± 0.16	0.451	0.5 ± 0.26	0.053
Inhaler medication possession ratio	0.42 ± 0.08	< 0.001	−0.05 ± 0.07	0.484	0.37 ± 0.12	0.002

Data are presented as mean ± standard deviation

*P-values were calculated through multivariable logistic regression analysis, including all variables

SE = standard error; D_LCO_ = diffusing capacity of the lungs for carbon monoxide; FEV_1_ = forced expiratory volume in 1 s

**Table 5:** Risk of any and frequent exacerbations according to smoking status

	Any exacerbation		Frequent exacerbations	
	aOR (95% CI)	*P*-value*	aOR (95% CI)	*P*-value*
Never-smoker (vs. ever-smoker)	0.46 (0.26–0.8)	0.006	0.28 (0.09–0.89)	0.03
FEV_1_, L	0.25 (0.17–0.37)	< 0.001	0.11 (0.51–0.22)	< 0.001
Blood neutrophil-to-lymphocyte ratio	1.1 (1.02–1.17)	0.011	1.01 (0.97–1.06)	0.684
Physician-diagnosed asthma	1.6 (1.05–2.44)	0.028	1.59 (0.77–3.26)	0.208
Interstitial lung disease	7.18 (1.48–34.95)	0.015	4.4 (0.83–23.23)	0.081
Inhaler medication possession ratio	2.13 (1.26–3.61)	0.005	3.87 (1.22–12.26)	0.022

*P-values were calculated through multivariable logistic regression analysis, including all variables

aOR = adjusted odds ratio; CI = confidence interval; FEV_1_ = forced expiratory volume in 1 s

Ex-smokers and current smokers had significantly higher risks of subsequent acute exacerbations during the follow-up period compared with never-smokers (aORs of 2.06 and 2.37, respectively) (Table S2). In particular, ex-smokers had a 3.97-fold higher risk of frequent exacerbations compared with never-smokers. When additional analysis was performed according to spirometric grade, the increased risk of subsequent acute exacerbations in ex-smokers and current smokers compared with never-smokers was significant in the GOLD grade 2 population (Table S3).

### Longitudinal changes in lung function according to smoking status

Evaluation of longitudinal changes in lung function adjusted for age, sex, and baseline lung function showed that never-smokers had a significantly slower decline in FEV_1_ compared with ever-smokers (− 15.7 ± 4.7 vs. −30.4 ± 2.9 mL/year, respectively; *P* = 0.03) (Table 6). Although the differences were not statistically significant, the annual rates of decline in FVC, FEV_1_/FVC ratio, and D_LCO_ also were slower in never-smokers than in ever-smokers.

**Table 6:** Longitudinal changes in lung function according to smoking status

Variables	Patients (*N* = 583)	Univariable		Multivariable*	
		β ± SE	*P*-value	β ± SE	*P*-value
FEV_1_, mL/year			0.234		0.03
Never-smoker	75	−18 ± 7.2		−15.7 ± 4.7	
Ever-smoker	508	−29.8 ± 4.1		−30.4 ± 2.9	
FVC, mL/year			0.249		0.107
Never-smoker	75	−19.1 ± 10.3		−18.2 ± 6.1	
Ever-smoker	508	−35 ± 6.3		−35.6 ± 4.5	
FEV_1_/FVC, ratio/year			0.566		0.195
Never-smoker	75	−0.2 ± 0.21		−0.16 ± 0.14	
Ever-smoker	508	−0.34 ± 0.09		−0.33 ± 0.06	
D_LCO_, %/year			0.668		0.544
Never-smoker	75	−0.09 ± 0.64		−0.06 ± 0.46	
Ever-smoker	508	−0.64 ± 0.24		−0.33 ± 0.19	

*Adjusted for age, sex, height, and baseline lung function (FEV_1_ or FVC or D_LCO_)

SE = standard error; FEV_1_ = forced expiratory volume in 1 s; FVC = forced vital capacity; D_LCO_ = diffusing capacity of the lungs for carbon monoxide

**Fig. 2:**
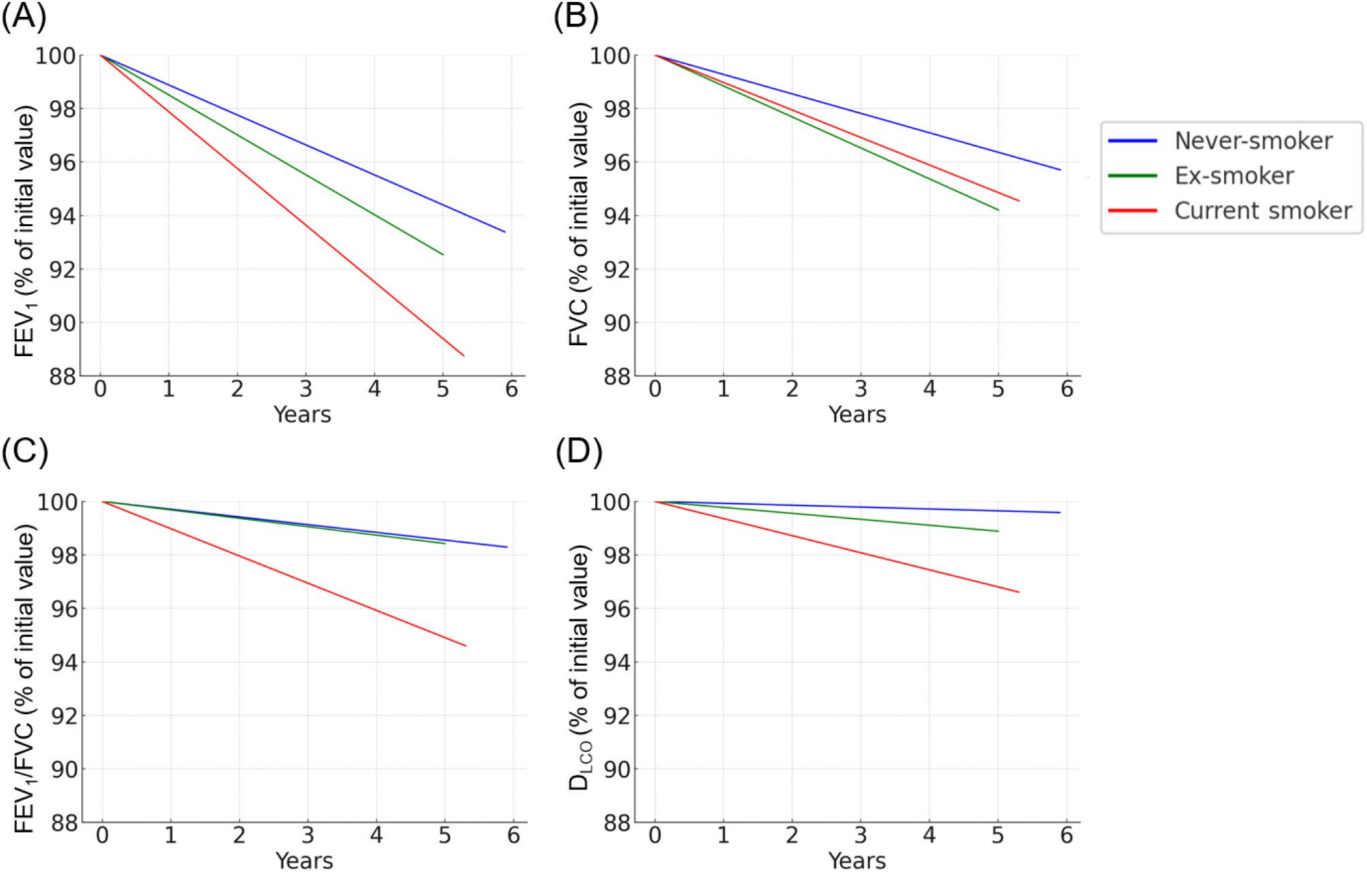
Comparative decline in lung function metrics by smoking status. This represents the longitudinal change in lung function according to smoking status. The changes in lung function during the study period were estimated using a linear mixed regression model based on baseline lung function and expressed as a percentage decline. Data was adjusted for age, sex, height, and baseline lung function. (**A**) FEV_1_. (**B**) FVC. (**C**) FEV_1_/FVC ratio. (**D**) D_LCO_. The blue line represents the never-smoker group, the green line represents the ex-smoker group, and the red line represents the current smoker group

Upon stratification according to smoking status, the annual rate of decline in FEV_1_ was fastest in current smokers (− 38.2 ± 4.8 mL/year), followed by ex-smokers (− 23.9 ± 3.6 mL/year) and then never-smokers (− 15.7 ± 4.7 mL/year) (Table S4). The decline in FEV_1_/FVC ratio, an indicator of obstructive disease progression, was similar between never-smokers and ex-smokers but significantly slower than in current smokers. FVC and D_LCO_ tended to decline more slowly in never-smokers than in ex-smokers and current smokers, although these differences were not statistically significant. The longitudinal changes in lung function according to smoking status during the study period are presented in Fig. 2. In the additional analysis with stratification according to spirometric grade, never-smokers showed a significantly slower annual decline in FEV_1_ compared with ex-smokers and current smokers within the GOLD grade 2 population (Table S5).

## Discussion

In this retrospective cohort, the prevalence of never-smokers among patients with COPD was 12.9%. Never-smokers with COPD were predominantly female; they had a higher prevalence of asthma, history of TB, TB-destroyed lung, and bronchiectasis but exhibited a lower prevalence of emphysema, one of the typical characteristics of smoking-induced COPD. The high predominance of women among never-smokers with COPD indicates a sex-related difference in COPD risk. Notably, sex disparities exist among risk factors for COPD, particularly in the prevalence of active and passive smoking histories, as well as exposure to biomass fuel [17]. In South Korea, where the present study was conducted, the Korea National Health and Nutrition Examination Survey (KNHANES) is performed on an annual basis to investigate the health and nutritional status of the Korean population. The results from the KNHANES 2007–2015, which included more than 73,000 participants, revealed that among men, the proportions of never-smokers and ever-smokers with a smoking history of ≥ 20 pack-years were 22.1% and 40.1%, respectively. Among women, however, these proportions were 90.8% and 1.9%, respectively [18]. This disparity indicates that sex is a major determinant of the proportion of smokers and highlights the need for a more detailed examination of risk factors other than smoking in women with COPD.

The present study demonstrated that asthma, a history of TB, TB-destroyed lung, and bronchiectasis are concurrent respiratory conditions associated with COPD in never-smokers. Asthma is characterized by chronic and repetitive airway inflammation due to airway hypersensitivity, and advanced asthma can acquire features of COPD through cumulative airway remodeling [19]. TB-destroyed lung and bronchiectasis may induce the development of infection-related COPD by reducing mucus clearance in the airways, leading to recurrent infections, airway inflammation, and small airway dysfunction [7, 20, 21]. In particular, TB-destroyed lung and bronchiectasis may lead to partial manifestations of restrictive ventilatory disorder due to destructive changes in the parenchyma or airways [22, 23]. These findings are consistent with the lower FVC observed in never-smokers with COPD relative to ever-smokers with COPD in our study. Furthermore, asthma, TB-destroyed lung, and bronchiectasis are conditions associated with small airway dysfunction independent of smoking, which might explain the lower FEF_25%–75%_ observed in never-smokers.

Our study also showed that never-smokers with COPD may develop exacerbations similar to those in ever-smokers, and some patients become frequent exacerbators. Acute exacerbation is an important prognostic factor associated with short- and long-term mortality risk in patients with COPD [24]. A study of the Canadian general population revealed that never-smokers with COPD had a higher prevalence of chronic respiratory symptoms such as cough, sputum production, exertional dyspnea, and wheezing relative to never-smokers without COPD [17]. Another study demonstrated that never-smokers with COPD had a higher incidence of lung cancer relative to ever-smokers without COPD [25]. These findings suggest that underlying risk factors predisposing never-smokers to COPD contribute to a worse prognosis than the absence of a history of smoking.

A major finding of this study is that never-smokers with COPD had a significantly lower risk of acute exacerbation during the follow-up period relative to ever-smokers with COPD. This result remained statistically significant even after adjustments for major prognostic factors such as lung function, comorbid diseases, and inhaler therapy. These findings are consistent with the results of a Copenhagen General Population Study cohort in Denmark, which demonstrated that never-smokers with COPD had lower inflammatory marker levels and a lower risk of hospitalization for COPD and pneumonia relative to ever-smokers with COPD [9]. The difference in the risk of acute exacerbation according to smoking status primarily suggests that smoking is a strong determinant of acute exacerbation and disease progression in patients with COPD. A recent large multinational study showed a positive proportional relationship between smoking intensity and COPD risk [7]. Factors related to COPD pathogenesis in never-smokers, such as genetic predisposition, environmental exposures, a history of respiratory infection, and comorbid respiratory diseases, may influence the distinct phenotype of COPD but have fewer impacts on rapid changes such as future acute exacerbations. In a study of pathological phenotypes, ever-smokers with COPD primarily showed emphysematous changes, whereas never-smokers with COPD primarily showed parenchymal and small airway fibrosis [26]. These results suggest that despite the similar lung function between never-smokers and ever-smokers with COPD, there are differences in clinical courses between these groups of patients.

Among patients with COPD in this study, the rate of FEV_1_ decline was significantly slower in never-smokers than in ever-smokers. The annual rate of FEV_1_ decline was significantly more rapid in the order of current smokers, ex-smokers, and never-smokers among the COPD population. Thus, the presence and persistence of a smoking history are strong determinants of lung function decline. The relatively slow decline of FEV_1_ in never-smokers with COPD may also be associated with comorbid respiratory conditions, such as TB-destroyed lung and bronchiectasis. These conditions may be associated with the development of COPD, as well as the decline in lung function that has already occurred; however, subsequent changes in lung function may not be clinically significant compared with the actual lung function decline because disease progression tends to be relatively indolent and less variable. These results are consistent with previous research findings that never-smokers with COPD experience a slower decline in lung function over time relative to ever-smokers with COPD; they also exhibit a pattern of small airway dysfunction, rather than emphysema [10]. In a study of long-term changes in lung function over a 15-year period across a cohort of Mexican patients with COPD, patients with biomass exposure-induced COPD exhibited a slower and more homogeneous decline in FEV_1_ over time relative to patients with smoking-related COPD [27]. Thus, in terms of lung function, disease progression may be slower in never-smokers with COPD than in ever-smokers with COPD. However, it is essential to consider the underlying factors and comorbidities contributing to COPD onset and progression when evaluating the prognosis in never-smokers with COPD.

In the present study, most never-smokers with COPD were using inhalers, and their use of inhalers did not significantly differ from the use among ever-smokers with COPD. Furthermore, never-smokers with COPD had a risk of acute exacerbations, although this risk was lower than the risk in ever-smokers with COPD; some never-smokers with COPD were frequent exacerbators. This finding indicates that never-smokers with COPD have a substantial need for pharmacological therapy. Our results suggest that never-smokers with COPD constitute a heterogeneous group with various clinical conditions other than smoking that can cause obstructive ventilatory disorders. However, most studies of pharmacological therapy for patients with COPD have predominantly targeted the ever-smoker population, often excluding patients with other respiratory conditions. Further research is warranted to develop personalized treatment strategies for COPD populations with diverse underlying conditions other than smoking.

This study had some limitations. First, the level of evidence was limited because of the retrospective study design, and the effectiveness of pharmacological therapy could not be evaluated because patients with more severe disease tended to receive more medication. Second, because this study targeted patients who underwent long-term follow-up at a tertiary referral hospital, there may have been patient selection-related bias. Especially, this study aimed to evaluate the long-term clinical course of a chronic disease, and therefore, patients with a follow-up period of less than one year were excluded. However, as a result, it is possible that patients in an extremely advanced stage of the disease, who experienced a rapid deterioration with a grave prognosis shortly after being diagnosed with COPD, were excluded. Third, the etiology of COPD can vary according to patients’ geographical locations and ethnicities. In South Korea, the smoking rate among men has historically been significantly higher than that of women. Consequently, in retrospective cohorts, the prevalence of smoking-related COPD tends to be markedly higher in men, a trend that is also observed in this study. This study is limited in its generalizability due to the study population being predominantly Asian and the presence of male predominance.

## Conclusions

Never-smokers with COPD had a significantly lower risk of acute exacerbations and a slower rate of decline in FEV_1_ relative to ever-smokers with COPD. In relation to these findings, we identified differences in sex, comorbid respiratory diseases, patterns of lung function, and radiological characteristics between the two groups. Future research focusing on more detailed evaluation and personalized treatment strategies for never-smokers with diverse etiologies of COPD is warranted.

## Electronic supplementary material

Below is the link to the electronic supplementary material.

Supplementary Material 1

## Data Availability

All data generated or analyzed during this study are included in this published article and its supplementary information files.

## Declarations

### Additional information

Supplementary tables can be found in the Supplementary Materials of the online article.
