# Supplementary material for: Distinct risks of exacerbation and lung function decline between never-smokers and ever-smokers with COPD
Source: BMC Pulm Med. 2025 Mar 28;25:138. doi: 10.1186/s12890-025-03604-1 (PMC11951795; doi:10.1186/s12890-025-03604-1)
Supplement: Supplementary file 1 — Supplementary Material 1 [file 12890_2025_3604_MOESM1_ESM.pdf]

Distinct risks of exacerbation and lung function decline between never-smokers and ever-smokers with COPD

ONLINE DATA SUPPLEMENT

Table S1. Baseline and clinical characteristics of the study population

	Never-smoker (n = 75)	Ex-smoker (n = 273)	Current smoker (n = 235)	P-value
Follow-up duration, year	5.9 ± 3.8	5 ± 3	5.3 ± 3.4	0.340*
Age, years	64.8 ± 11.3	68.8 ± 8.5	63.7 ± 9.1	<0.001*
Female sex, n (%)	38 (50.7)	6 (2.2)	11 (4.7)	<0.001†
Body mass index, kg/m^2	22.3 ± 3.2	22.4 ± 3.4	22.2 ± 3.4	0.44*
Smoking intensity, pack years	0 ± 0	42.1 ± 26.2	42.6 ± 20.5	<0.001*
Charlson Comorbidity Index	1.5 ± 1	1.6 ± 1.0	1.6 ± 1.1	0.925*
History of tuberculosis, n (%)	33 (44)	73 (26.7)	49 (20.9)	<0.001†
History of NTM lung disease, n (%)	3 (4)	2 (0.7)	4 (1.7)	0.123†
Physician-diagnosed asthma, n (%)	28 (37.3)	73 (26.7)	51 (21.7)	0.026†
Baseline lung function				
FEV_1, L	1.4 ± 0.5	1.6 ± 0.5	1.8 ± 0.6	<0.001*
FEV_1, % predicted	62.3 ± 18	65.5 ± 19.2	67.8 ± 18.8	0.069*
FVC, L	2.5 ± 0.7	3.2 ± 0.8	3.4 ± 0.8	<0.001*
FVC, % predicted	78.8 ± 17.2	88.3 ± 17.7	91.0 ± 17.6	<0.001*
FEV_1/FVC, %	55.3 ± 10	50.8 ± 11.9	52.0 ± 11.3	0.016*
$FEF_{25–75\%}$, % predicted	24.9 ± 9.8	27.1 ± 11.5	28.8 ± 11.7	0.039*
D_{LCO}, mL/mmHg/min	14.2 ± 4.1	13.1 ± 4.03	14 ± 4.5	0.078*
D_{LCO}, % predicted	85.6 ± 22.7	80.9 ± 20.8	81.3 ± 21.7	0.101*
Positive bronchodilator response, n (%)	1 (1.3)	31 (11.4)	37 (15.7)	0.003†
Laboratory findings				
Blood neutrophil-to-lymphocyte ratio	4.6 ± 9.2	3.9 ± 4.1	3.2 ± 2.7	0.049*
Blood eosinophil count, × 10^3/L	221.6 ± 274.8	214.3 ± 226.1	237.1 ± 242.7	0.2*
Blood eosinophil count > 300 × 10^3/L, n (%)	14 (18.7)	59 (21.6)	54 (23)	0.73†
Baseline CT findings				
Emphysema, n (%)	15 (20)	214 (78.4)	192 (81.7)	<0.001†
Tuberculous-destroyed lung, n (%)	18 (24)	24 (8.8)	17 (7.2)	<0.001†
Bronchiectasis, n (%)	52 (69.3)	137 (50.2)	89 (37.9)	<0.001†
Interstitial lung disease, n (%)	1 (1.3)	6 (2.2)	7 (3)	0.689†
Inhaler use, n (%)	74 (98.7)	257 (94.1)	210 (89.4)	0.013†
Inhaler medication possession ratio	0.65 ± 0.3	0.68 ± 0.35	0.59 ± 0.37	0.003*

Data are presented as mean ± standard deviation or n (%).

*P-values were calculated using the **t-test** for continuous variables.

†P-values were calculated using the **chi-square test** (or Fisher's exact test where applicable) for categorical variables.

CT = computed tomography; D_{LCO} = diffusing capacity of the lungs for carbon monoxide; $FEF_{25–75\%}$ = forced expiratory flow between 25% and 75% of vital capacity; FEV_1 = forced expiratory volume in 1 s; FVC = forced vital capacity; NTM = nontuberculous mycobacteria

Table S2. Risk of any and frequent exacerbations according to smoking status

	Any exacerbation		Frequent exacerbation	
	aOR (95% CI)	P-value*	aOR (95% CI)	P-value*
Smoking status				
Never-smoker	1 (ref)		1 (ref)	
Ex-smoker	2.06 (1.16−3.66)	0.014	3.97 (1.25−12.64)	0.019
Current smoker	2.37 (1.3−4.32)	0.005	2.79 (0.82−9.51)	0.102
FEV_1, L	0.25 (0.17−0.37)	<0.001	0.11 (0.05−0.23)	<0.001
Blood neutrophil-to-lymphocyte ratio	1.1 (1.02−1.18)	0.011	1.01 (0.96−1.06)	0.723
Physician-diagnosed asthma	1.62 (1.06−2.47)	0.025	1.56 (0.76−3.21)	0.228
Interstitial lung disease	7.14 (1.47−34.7)	0.015	4.57 (0.86−24.41)	0.075
Inhaler medication possession ratio	2.17 (1.28−3.67)	0.004	3.55 (1.12−11.3)	0.032

*P-values were calculated through multivariable logistic regression analysis, including all variables.

Table S3. Risk of any and frequent exacerbations according to smoking status and GOLD spirometric grade

	Any exacerbation		Frequent exacerbation	
	aOR (95% CI)	P-value	aOR (95% CI)	P-value
GOLD grade 1 (n=155)				
Never vs. ever-smoker	1.65 (0.46−5.94)	0.446	−	−
Never-smoker	1 (ref)		−	
Ex-smoker	0.57 (0.15−2.14)	0.406	−	−
Current smoker	0.67 (0.17−2.62)	0.562	−	−
GOLD grade 2 (n=309)				
Never vs. ever-smoker	0.37 (0.18−0.76)	0.007	0.32 (0.07−1.57)	0.161
Never-smoker	1 (ref)		1 (ref)	
Ex-smoker	2.63 (1.24−5.6)	0.012	3.23 (0.64−16.3)	0.157
Current smoker	2.82 (1.28−6.2)	0.01	2.91 (0.52−16.42)	0.226
GOLD grade 3 (n=96)				
Never vs. ever-smoker	0.32 (0.07−1.37)	0.125	0.39 (0.04−3.59)	0.406
Never-smoker	1 (ref)		1 (ref)	
Ex-smoker	2.67 (0.58−12.27)	0.208	2.8 (0.29−26.81)	0.373
Current smoker	4.32 (073−25.54)	0.106	2.22 (0.21−23.28)	0.508
GOLD grade 4 (n=23)				
Never vs. ever-smoker	−	−	0.64 (0.04−10.29)	0.751
Never-smoker	−		1 (ref)	
Ex-smoker	−	−	2.64 (0.16−43.38)	0.497
Current smoker	−	−	0.54 (0.02−14.07)	0.709

All analyses were adjusted for FEV_1, blood neutrophil-to-lymphocyte ratio, physician-diagnosed asthma, interstitial lung disease, and inhaler medication possession ratio

Table S4. Longitudinal changes in lung function according to smoking status

Variables	Patients (N = 583)	Univariable		Multivariable*	
		β ± SE	P-value	β ± SE	P-value
FEV_1, mL/year			0.06		0.006
Never-smoker	75	−18.0 ± 7.2		−15.7 ± 4.7	
Ex-smoker	273	−22.7 ± 5		−23.9 ± 3.6	
Current smoker	235	−38.5 ± 6.6		−38.2 ± 4.8	
FVC, mL/year			0.451		0.261
Never-smoker	75	−19.1 ± 10.3		−18.2 ± 6.1	
Ex-smoker	273	−33 ± 8.1		−37.1 ± 5.8	
Current smoker	235	−38.7 ± 9.8		−35 ± 7.3	
FEV_1/FVC, ratio/year			0.067		0.008
Never-smoker	75	−0.2 ± 0.21		−0.16 ± 0.14	
Ex-smoker	273	−0.15 ± 0.12		−0.16 ± 0.09	
Current smoker	235	−0.55 ± 0.13		−0.53 ± 0.09	
D_{LCO}, %/year			0.371		0.607
Never-smoker	75	−0.09 ± 0.64		−0.06 ± 0.46	
Ex-smoker	273	−0.38 ± 0.35		−0.18 ± 0.27	
Current smoker	235	−0.9 ± 0.33		−0.52 ± 0.27	

*Adjusted for age, sex, height, and baseline lung function (FEV_1 or FVC or D_{LCO})

Table S5. Longitudinal changes in FEV_1 according to smoking status and GOLD spirometric grade

FEV_1, mL/year	Patients (N = 583)	Univariable		Multivariable*	
		β ± SE	P-value	β ± SE	P-value
GOLD grade 1			0.534		0.37
Never-smoker	13/155	−60.4 ± 17.4		−61.4 ± 7.8	
Ex-smoker	72/155	−38.1 ± 10.1		−42.1 ± 7.9	
Current smoker	70/155	−56.7 ± 14		−58.2 ± 11.3	
GOLD grade 2			0.077		0.001
Never-smoker	45/309	−9 ± 9.6		−1.1 ± 6.1	
Ex-smoker	137/309	−24.4 ± 5.8		−26.2 ± 4.2	
Current smoker	127/309	−36 ± 7.1		−38.7 ± 5.7	
GOLD grade 3			0.482		0.353
Never-smoker	11/96	−3.1 ± 13.2		1.1 ± 9.2	
Ex-smoker	56/96	5.7 ± 11		7.6 ± 9.1	
Current smoker	29/96	−16,1 ± 15.5		−15.2 ± 14.3	
GOLD grade 4			0.495		0.58
Never-smoker	6/23	15.3 ± 16.3		9.8 ± 13.2	
Ex-smoker	8/23	41.5 ± 15.7		36.3 ± 13.8	
Current smoker	9/23	0.41 ± 22.7		0.33 ± 24.5	

*Adjusted for age, sex, height, and baseline lung function (FEV_1 or FVC or D_{LCO})
